# Differential Disease Susceptibilities in Experimentally Reptarenavirus-Infected Boa Constrictors and Ball Pythons

**DOI:** 10.1128/JVI.00451-17

**Published:** 2017-07-12

**Authors:** Mark D. Stenglein, David Sanchez-Migallon Guzman, Valentina E. Garcia, Marylee L. Layton, Laura L. Hoon-Hanks, Scott M. Boback, M. Kevin Keel, Tracy Drazenovich, Michelle G. Hawkins, Joseph L. DeRisi

**Affiliations:** a Department of Microbiology, Immunology, and Pathology, College of Veterinary Medicine and Biomedical Sciences, Colorado State University, Fort Collins, Colorado, USA; b Department of Medicine and Epidemiology, School of Veterinary Medicine, University of California, Davis, Davis, California, USA; c Chan Zuckerberg Biohub and the Department of Biochemistry & Biophysics, University of California, San Francisco, San Francisco, California, USA; d Department of Biology, Dickinson College, Carlisle, Pennsylvania, USA; e Department of Pathology, Microbiology & Immunology, School of Veterinary Medicine, University of California, Davis, Davis, California, USA; University of Texas Southwestern Medical Center

**Keywords:** arenavirus, inclusion body disease, pathogenesis, reptarenavirus, veterinary pathogens

## Abstract

Inclusion body disease (IBD) is an infectious disease originally described in captive snakes. It has traditionally been diagnosed by the presence of large eosinophilic cytoplasmic inclusions and is associated with neurological, gastrointestinal, and lymphoproliferative disorders. Previously, we identified and established a culture system for a novel lineage of arenaviruses isolated from boa constrictors diagnosed with IBD. Although ample circumstantial evidence suggested that these viruses, now known as reptarenaviruses, cause IBD, there has been no formal demonstration of disease causality since their discovery. We therefore conducted a long-term challenge experiment to test the hypothesis that reptarenaviruses cause IBD. We infected boa constrictors and ball pythons by cardiac injection of purified virus. We monitored the progression of viral growth in tissues, blood, and environmental samples. Infection produced dramatically different disease outcomes in snakes of the two species. Ball pythons infected with Golden Gate virus (GoGV) and with another reptarenavirus displayed severe neurological signs within 2 months, and viral replication was detected only in central nervous system tissues. In contrast, GoGV-infected boa constrictors remained free of clinical signs for 2 years, despite high viral loads and the accumulation of large intracellular inclusions in multiple tissues, including the brain. Inflammation was associated with infection in ball pythons but not in boa constrictors. Thus, reptarenavirus infection produces inclusions and inclusion body disease, although inclusions *per se* are neither necessarily associated with nor required for disease. Although the natural distribution of reptarenaviruses has yet to be described, the different outcomes of infection may reflect differences in geographical origin.

**IMPORTANCE** New DNA sequencing technologies have made it easier than ever to identify the sequences of microorganisms in diseased tissues, i.e., to identify organisms that appear to cause disease, but to be certain that a candidate pathogen actually causes disease, it is necessary to provide additional evidence of causality. We have done this to demonstrate that reptarenaviruses cause inclusion body disease (IBD), a serious transmissible disease of snakes. We infected boa constrictors and ball pythons with purified reptarenavirus. Ball pythons fell ill within 2 months of infection and displayed signs of neurological disease typical of IBD. In contrast, boa constrictors remained healthy over 2 years, despite high levels of virus throughout their bodies. This difference matches previous reports that pythons are more susceptible to IBD than boas and could reflect the possibility that boas are natural hosts of these viruses in the wild.

## INTRODUCTION

Inclusion body disease (IBD) has been a vexing problem in captive snake collections for several decades (1). Classic clinical signs of IBD include neurological signs, regurgitation, and secondary bacterial infections, including stomatitis and pneumonia (2). More recently, several cases of lymphoproliferative disorders have been associated with IBD in boa constrictors (2–5). Different clinical outcomes have been described for boas and pythons, with pythons reportedly experiencing a shorter, more severe, and more central nervous system (CNS)-involved disease course (1, 2, 6, 7). Passage experiments demonstrated IBD to be transmissible, but the etiological agent remained elusive, until the recent identification and isolation of arenaviruses from snakes diagnosed with IBD (1, 7–10).

Two major groups of arenaviruses (family Arenaviridae) have been identified: those that infect mammals (genus Mammarenavirus) and those that infect snakes (genus Reptarenavirus) (11–13). Arenaviruses share a number of common characteristics, including a bisegmented single-stranded RNA genome with two genes on each of the small (S) and large (L) genome segments in an ambisense orientation (11, 12). One possibly distinguishing feature of reptarenaviruses is that simultaneous infection by multiple viruses is common in captive snakes (14–16). Whether this is true in wild snakes is unclear, and in fact, there is no published information about the natural hosts of reptarenaviruses, although IBD has been described in a number of captive snakes of a number of species worldwide, and reptarenaviruses have been identified in snakes on multiple continents (2, 6, 8–10, 14, 17–19).

There is strong indirect evidence that reptarenaviruses cause IBD. First, reptarenavirus RNA detection and viral recovery are correlated with IBD diagnosis (8–10, 14). Second, cytoplasmic inclusions, the historical diagnostic hallmark of IBD, contain reptarenavirus nucleoprotein (NP) (10, 14, 20, 21). Third, several independent metagenomic next-generation sequencing (NGS) studies have not identified other candidate etiological agents (8–10, 14, 15). Nevertheless, apparently healthy snakes can be infected with reptarenaviruses and even harbor inclusion bodies (22). In fact, 5 of the first 6 apparently healthy boa constrictors that we obtained initially for this study proved to be already infected with reptarenavirus. Clearly, infection does not always or immediately produce disease. Therefore, the purpose of this study was to determine whether reptarenavirus infection can cause IBD, as a formal demonstration of disease causality and as a step toward identification of viral and host determinants of pathogenicity, and to study the outcome of reptarenavirus infection in snakes of multiple species.

We therefore experimentally infected boa constrictors (Boa constrictor) and ball pythons (Python regius) with reptarenaviruses. We monitored infected snakes and uninfected controls. We periodically collected blood samples and tissue biopsy samples to monitor virus replication and collected environmental samples to assess possible mechanisms of transmission. Infected boa constrictors remained subclinical over 2 years, despite high and disseminated viral loads and the accumulation of inclusion bodies. In contrast, infected ball pythons exhibited severe neurological signs within 2 months after infection, with viral nucleic acid and protein being detected only in the brain.

## RESULTS

**FIG 1:**
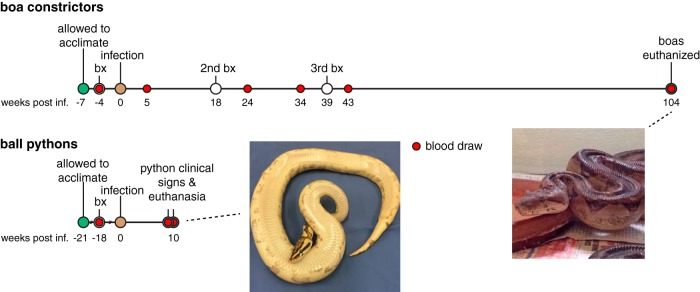
Timeline of experimental reptarenavirus infection of boa constrictors and ball pythons. The times of collection of the pre- and postinfection biopsy samples (bx) and blood samples tested are indicated. weeks post inf., weeks postinfection. (Insets) Images of a representative infected boa constrictor and representative infected ball python at the end of their respective study periods.

To confirm the absence of preexisting virus infection in the snakes to be used for experimental infections, blood, lung, and liver biopsy samples were collected and examined histologically and tested for reptarenavirus RNA by quantitative reverse transcription-PCR (qRT-PCR) and metagenomic NGS. Five of the first six boa constrictors that we obtained initially tested positive for viral RNA (designated boas A to F). Three additional boa constrictors from a closed collection tested negative and were used for infection studies (boas G to I). We infected snakes G and H with 4 × 10^5^ fluorescent focus-forming units (FFU) of Golden Gate virus (GoGV), a prototypic reptarenavirus, by intracardiac injection (8). Virus had been purified from the supernatant of infected boa constrictor JK cells (8). The third snake (boa I) was mock infected. Following inoculation, snakes were monitored and blood samples and liver and lung biopsy samples were periodically collected. Feces, urates, and shed skin samples were also collected to assess possible routes of virus shedding (Fig. 1).

Similar to the boa constrictors, four ball pythons were obtained and confirmed to be negative for preexisting virus infection (pythons J to M). One ball python (python L) was infected with 4 × 10^5^ FFU of GoGV, and a second one (python M) was coinfected with 2 × 10^5^ FFU of GoGV and 2 × 10^5^ FFU of a reptarenavirus isolated from a boa constrictor that had exhibited stomatitis and anorexia and had been euthanized and diagnosed postmortem with IBD (snake 37 in the study described in reference 14). Our rationale for coinfecting python M was 2-fold: to assess the pathogenic potential of genetically diverse reptarenaviruses (the S segments of the two viruses share ∼74% pairwise nucleotide identity) and to conduct a preliminary investigation of multiple reptarenavirus infection, which is surprisingly common in captive snakes (14, 15). Feces, urates, and shed skin samples were collected.

None of the boa constrictors developed clinical signs during the 2-year experiment. All snakes behaved normally and gained weight equivalently. The three boa constrictors were euthanized at the end of the study period, 24 months postinoculation. Complete postmortem examinations were performed, and tissues were collected from all major organs for pathological examination and virus detection.

In contrast, ball pythons exhibited severe clinical signs within ∼2 months of infection. At 65 days postinfection, python M developed an acute onset of neurological signs characteristic of IBD, including lethargy, abnormal posture, and failure to recover from dorsal recumbency (Fig. 1, inset; see also Video S1 in the supplemental material). This snake was immediately euthanized. Three days later (68 days postinfection), python L was observed to have focal dermatitis of unknown etiology on its right side. Further evaluation revealed that the snake had paralysis of the caudal 80% of its body and did not respond to hypodermic needle insertions in that area. It was unclear whether the dermatitis was related to infection. The snake was immediately euthanized. The control ball pythons did not display any clinical signs and were euthanized at day 68 as well. Complete postmortem examinations of the ball pythons were performed, but no antemortem biopsy samples were collected because of rapid disease onset.

**FIG 2:**
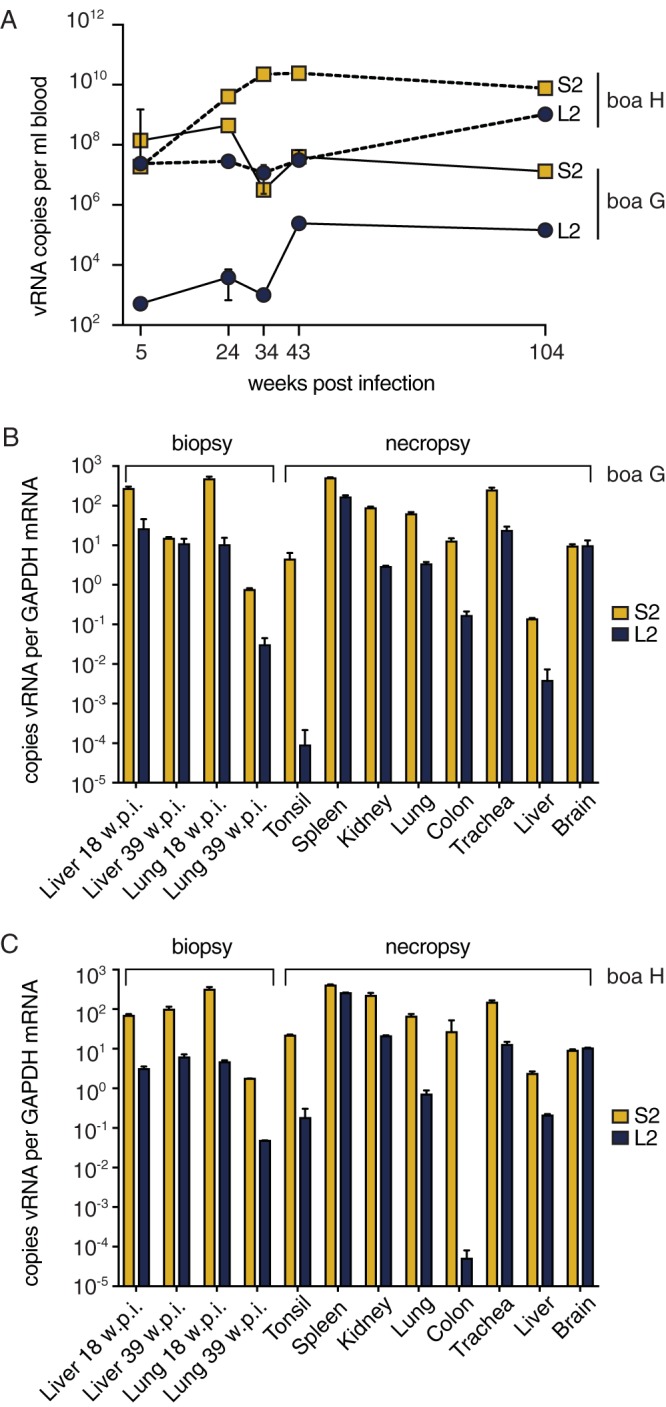
Boa constrictors had persistently high viral loads in all tissues. Viral RNA (vRNA) levels were quantified by qRT-PCR. (A) Blood viral RNA levels. (B and C) Tissue viral RNA levels for snake G (B) and snake H (C). Samples from the uninfected boa constrictor were negative. w.p.i., weeks postinfection; S2 and L2, viral genome segment genotypes.

**FIG 3:**
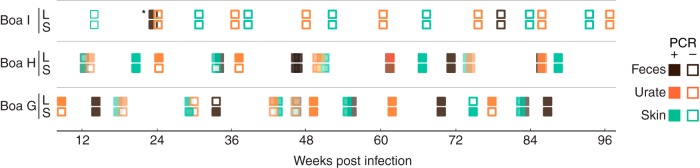
Viral RNA is detectable in feces, urates, and shed skin from infected boa constrictors. Viral RNA was detected by qRT-PCR. Viral RNA was not detected in any fecal, urate, or skin sample collected from ball pythons. *, the positive result for this fecal sample from the control snake may have resulted from sample mislabeling; no other sample from this control animal ever tested positive.

We used qRT-PCR to measure viral RNA levels in tissues. Despite the absence of clinical signs in the boa constrictors, high-level systemic virus replication was evident. Viral RNA was detectable in blood samples throughout infection at concentrations that ranged from 10^3^ to 10^10^ genome equivalents per ml of blood (Fig. 2A). Viral RNA was detected in antemortem liver biopsy samples and in all tissues assayed postmortem: liver, lung, tonsil, spleen, kidney, colon, trachea, and brain (Fig. 2B and C). The levels of viral RNA varied but reached concentrations exceeding 100-fold the copy number of the glyceraldehyde-3-phosphate dehydrogenase (GAPDH) control mRNA. Viral RNA was also detected in feces, urates, and skin shed from boa constrictors collected throughout the 2-year infection (Fig. 3). Attempts to isolate virus from these environmental samples were unsuccessful, perhaps because the samples may not have been processed or stored in a manner that preserved infectivity. These results show that boa constrictors support high reptarenavirus loads in the absence of clinical signs and shed detectable viral RNA in feces, urates, and skin.

**FIG 4:**
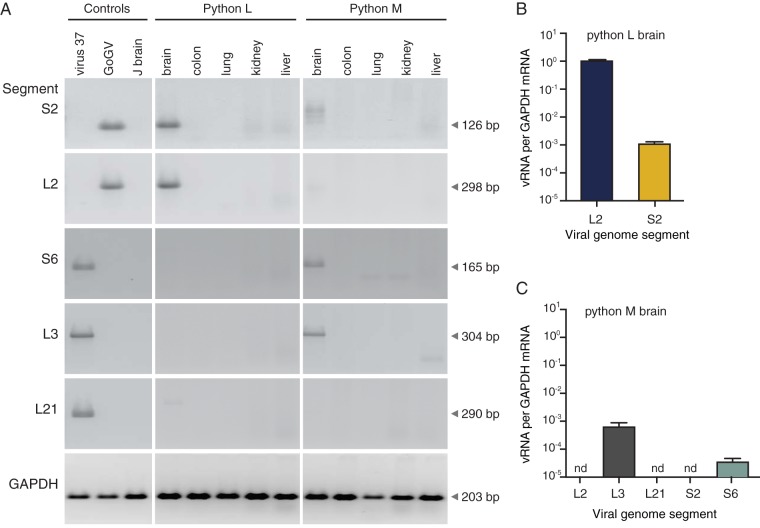
Viral RNA was detectable in infected ball python brains. Viral and cellular RNA levels were quantified by qRT-PCR. (A) Reptarenavirus RNA was detected in brain but not other tissues. (B and C) Viral RNA levels were normalized to the levels of GAPDH mRNA in ball python L (B) and M (C) brains. Samples from uninfected snakes were negative. Controls were the virus 37 inoculum (virus 37), the GoGV inoculum (GoGV), and uninfected python J brain (J brain). nd, not detected.

In ball pythons, viral RNA was detected only in the central nervous system of both infected snakes but not in other tissues tested (blood, colon, liver, lung, and kidney; Fig. 4A). Segments of genotypes S2 and L2 were detected in the brain of snake L (Fig. 4B). Segments of genotypes S6 and L3 were detected by qRT-PCR in the brain of snake M, which had been coinfected with GoGV (S2/L2) and snake 37 virus (genotype S6/L3/L21) (Fig. 4C). We created shotgun NGS libraries from total RNA extracted from the brains of the two infected ball pythons to confirm the absence of other organisms that could be responsible for neurological signs and did not identify other candidate pathogen sequences. Viral RNA was not detected in feces, urates, or shed skin collected from the ball pythons.

**FIG 5:**
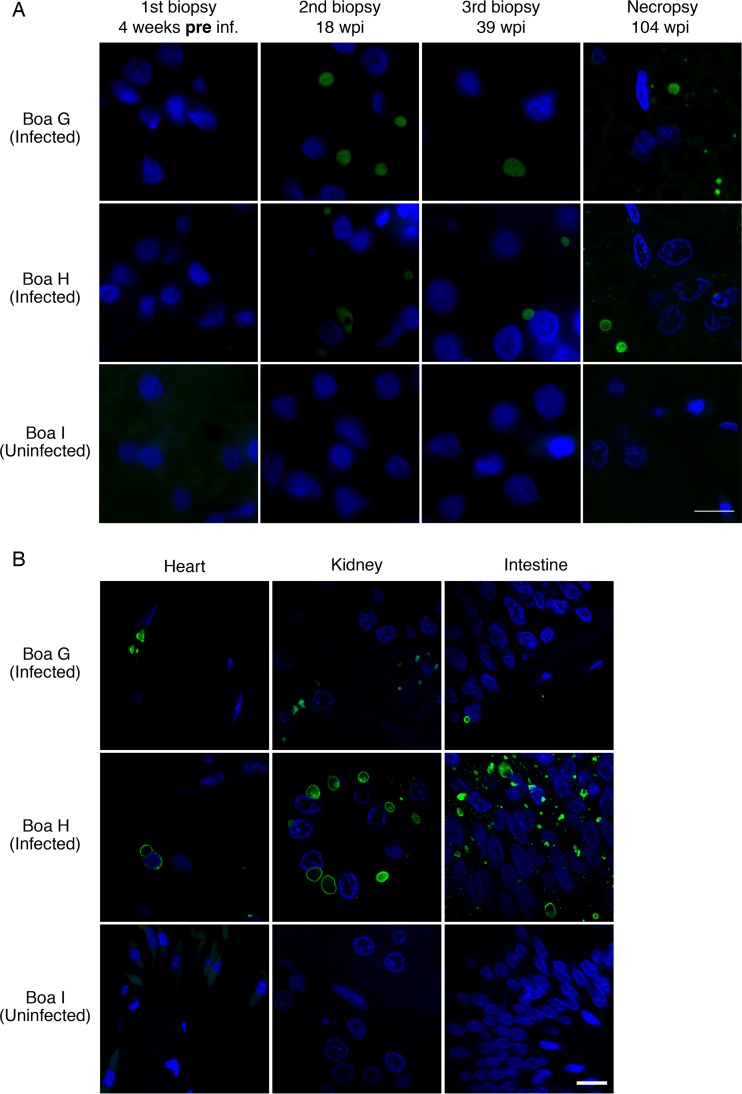
Reptarenavirus nucleoprotein-positive inclusions were detected in tissues of infected boa constrictors throughout infection. (A) Biopsy and necropsy liver sections from infected and uninfected boa constrictors were stained with anti-NP antibody (green) and DAPI (blue). (B) Necropsy heart, kidney, and intestine sections were stained as described in the legend to panel A. Bars = 10 μm.

**FIG 6:**
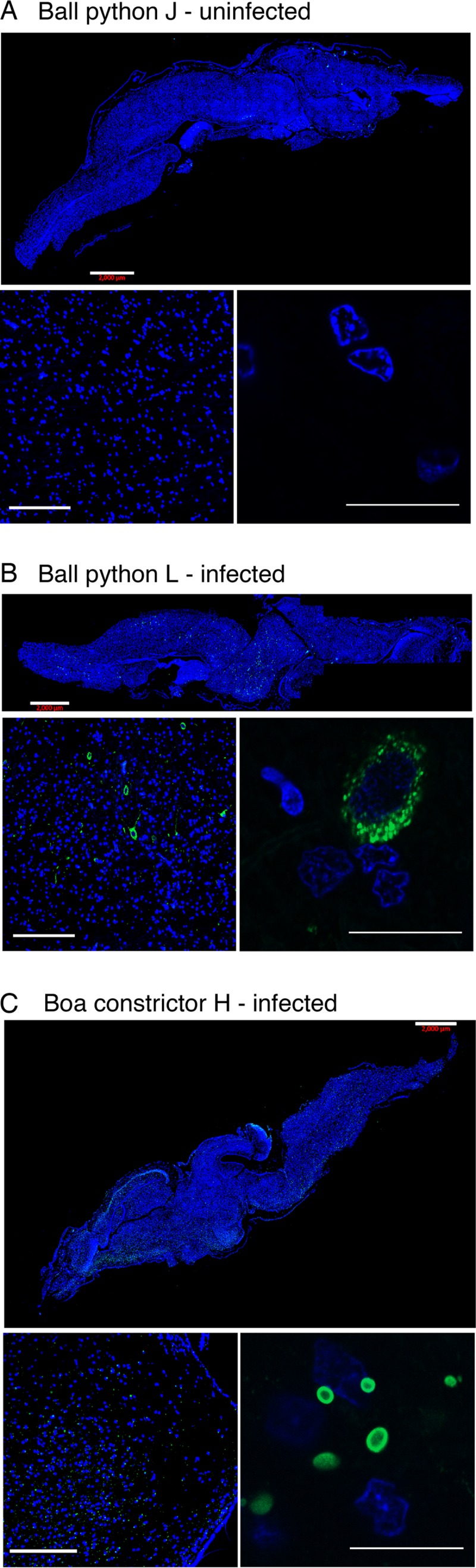
Reptarenavirus nucleoprotein detected in the brains of infected snakes. Brain sections were stained with anti-NP antibody (green) and with DAPI (blue). Sections were obtained from uninfected ball pythons J (A), infected ball python L (B), and infected boa constrictor H (C). The top, lower left, and lower right panels display increasingly zoomed images of the same sections, in which the bars are 2,000, 200, and 20 μm, respectively. Contrast speckled cytoplasmic staining in infected ball python cells (B) with inclusions in infected boa constrictor cells (C).

We used fluorescence microscopy with an antibody raised against a peptide from GoGV NP to visualize viral protein in tissues. In tissues from infected boa constrictors at necropsy, we observed large cytoplasmic NP-positive inclusions in every tissue examined: heart, intestinal, liver, kidney, and brain (Fig. 5 and 6). Viral inclusions were also apparent in the liver biopsy samples taken from both infected boas at 16 weeks and 32 weeks postinfection but were not evident in tissue samples collected preinfection (Fig. 5).

**FIG 7:**
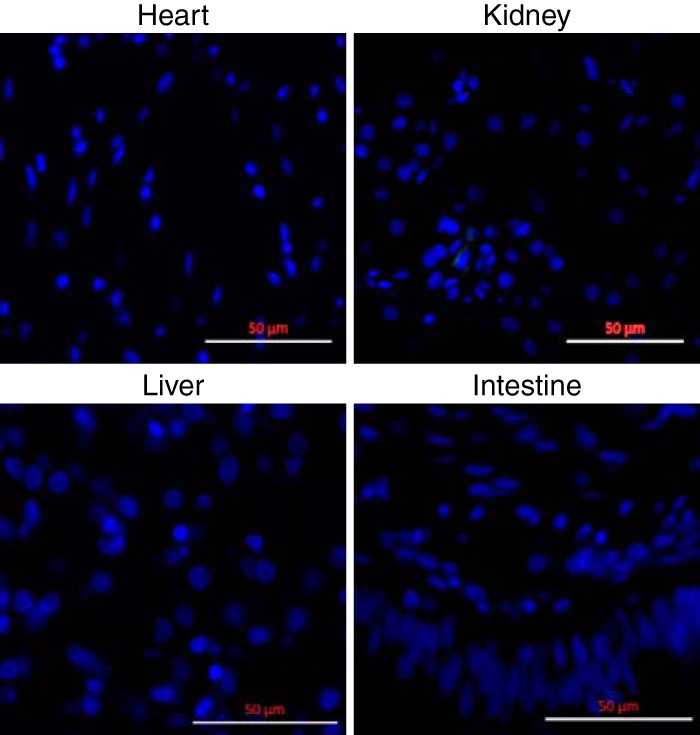
Reptarenavirus nucleoprotein was not detected in non-CNS tissues of infected ball pythons. Necropsy tissues from infected ball python L were stained with anti-NP antibody (green) and DAPI (blue). These images are representative of negative staining of all non-CNS tissues from all ball pythons. Bars = 50 µm.

In infected ball pythons, we did not detect NP-staining inclusions in any tissues except for brain (Fig. 6 and 7). Anti-NP antibody staining was present in brain cells of ball python L, but in contrast to the inclusions found in boa constrictor tissues, including brain, the staining appeared to be diffusely cytoplasmic (compare Fig. 6B and C). For the brain of python M, anti-NP staining was observed, but the fixed slices from python M were not of sufficient quality for staining by DAPI (4′,6-diamidino-2-phenylindole), limiting our ability to characterize infection in this specimen. Anti-NP staining was absent from all other ball python necropsy tissues, including heart, kidney, intestinal, and liver (Fig. 7).

**FIG 8:**
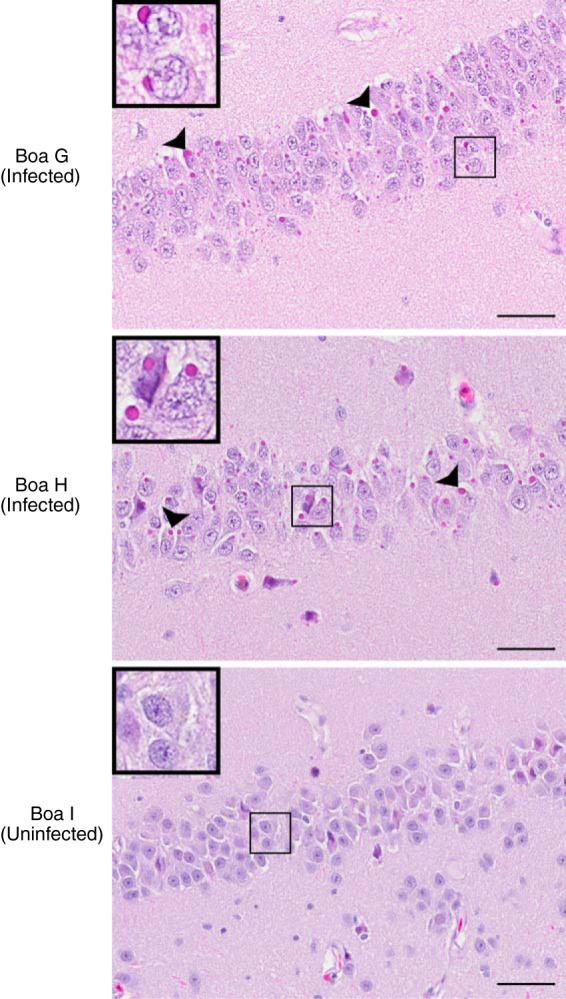
Inclusions were evident in infected boa constrictor brains. Images of hematoxylin and eosin (H&E)-stained brain sections from the indicated boa constrictors are shown. Infected boa constrictors (boas G and H) had numerous, brightly eosinophilic, cytoplasmic viral inclusion bodies (arrowheads) within neuronal cell bodies and glial cells of the brain. Similar inclusions were found within cells of nearly every organ examined. No inflammation was associated with the inclusions. The uninfected boa constrictor (boa I) did not have inclusions. (Insets) Magnified views of the boxed regions. Bars = 50 μm.

Gross and histopathological examinations were performed on euthanized snakes. In both infected and control boa constrictors, gross lesions were mild or considered incidental. The most notable histological change in boa constrictors was the presence of large eosinophilic inclusions in tissues throughout the body, and in some tissues the majority of cells were affected. Most (boa H) to virtually all (boa G) neurons in the brain and spinal cord had sharply demarcated inclusions (Fig. 8). Inclusions were most dense in the retina, neurons, bile duct epithelium, ductuli efferentes, exocrine pancreas, stomach, and kidney. Inclusion bodies were common in lymphocytes of all tissues in infected boa H but not in boa G. Inclusions were also noted in peripheral ganglia, the optic nerve, seminiferous tubules, oviductal glands, adrenal glands, harderian glands, small intestine, respiratory epithelium, pulmonary smooth muscle, cardiomyocytes, hepatocytes, and multiple vessels. Inclusions were absent in the uninfected boa constrictor. Despite the abundant inclusions, little inflammation was observed, and that which was observed was not considered related to infection.

**FIG 9:**
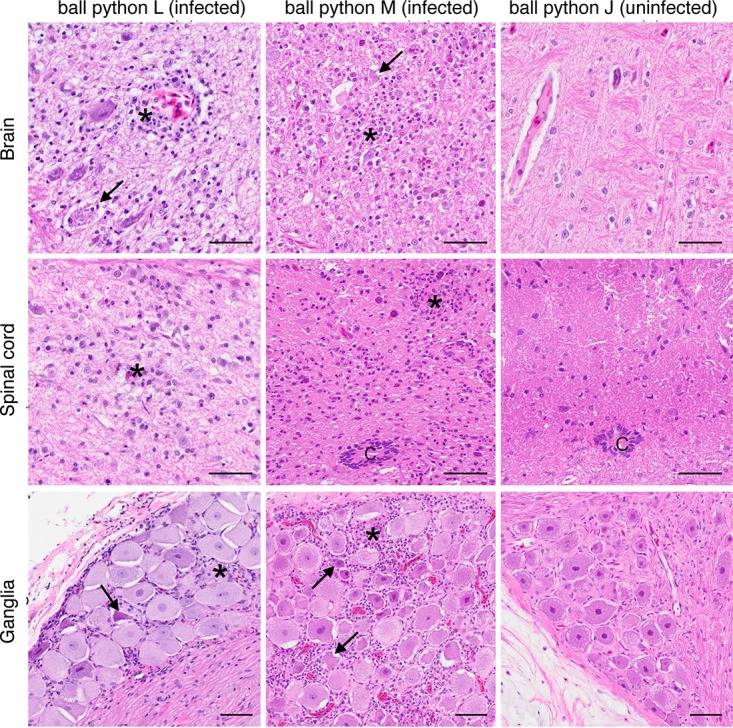
Inflammation in infected ball python central nervous system tissues. Arenavirus-infected pythons (pythons L and M) had moderate lymphocytic, histiocytic, and granulocytic inflammation (asterisks) within the brain, spinal cord, and ganglia. Necrotic neurons were occasionally seen (arrows). No inflammation was detected in the uninfected pythons. C, central canal. H&E-stained tissue section. Bars = 50 μm.

**FIG 10:**
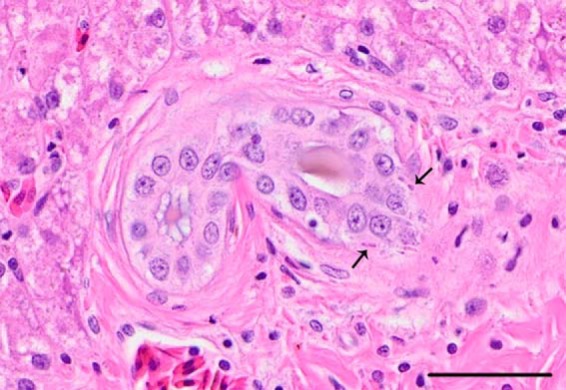
Bile duct inclusions in ball python M. Small eosinophilic cytoplasmic inclusions (arrows) were seen in rare bile duct epithelial cells of the infected ball python M. H&E-stained tissue section. Bar = 50 μm.

Pathological examinations of ball pythons revealed a picture markedly different from that in boas, characterized by central nervous system inflammation and a general lack of obvious inclusions. No gross lesions were detected in python M. Regionally extensive dermatitis, the cause and significance of which were unknown, was found in python L. The most significant histopathologic findings were inflammatory changes in the brain, spinal cord, and ganglia of both infected ball pythons (Fig. 9). Infected pythons had mild to moderate lymphocytic encephalitis; lymphocytic ganglioneuritis; and lymphocytic, histiocytic, and granulocytic meningomyelitis. Neuronal necrosis and neuronophagia were also present (Fig. 9). At the site of the dermatitis observed on infected python L, multiple variably sized foci of necrosis with heterophilic infiltrates were observed. Other histological changes included moderate lymphocytolysis in multiple lymphoid organs and minimal lymphocytic biliary dochitis (python M). In infected ball pythons, the presence of inclusion bodies was equivocal, with possible viral inclusions being observed in neurons and rare bile ducts of infected python M (Fig. 10). In both pythons, multiple types of epithelial cells had eosinophilic granular material within the cytoplasm. Although these granules were suggestive of inclusions, the material was generally more lightly stained and indistinct compared to typical inclusions of IBD. In control snakes (snakes J and K), no significant gross or microscopic lesions were observed.

## DISCUSSION

Reptarenaviruses were first identified in cases of IBD, and substantial but indirect evidence suggested that reptarenavirus infection causes disease (8–10, 14, 15). While infection of both boa constrictors and ball pythons resulted in the presence of detectable viral replication, we noted a stark contrast between the outcomes in the two types of snakes. During 2 years of infection, boa constrictors maintained high levels of viremia (10^3^ to 10^10^ viral copies per ml of blood) and accumulated widespread intracytoplasmic inclusions. Despite the high viral load and numerous inclusion bodies, boas did not display overt clinical signs by the time that they were euthanized, and there was a notable absence of inflammation. In contrast, infection of ball pythons produced dramatic clinical signs over the course of only ∼60 days. In pythons, inclusions were extremely rare, virus was detected only in the CNS, and pronounced inflammation was observed. These findings are by and large concordant with those of two IBD transmission experiments in Burmese pythons and boa constrictors that were conducted prior to the identification of reptarenaviruses (1, 7). Additional studies will be required to untangle the factors underlying this species-specific clinical outcome. It is also likely that not all snakes (even of the same species) respond identically to infection, and additional studies using larger numbers of infected snakes could reveal variability in clinical outcomes that our study, with its relatively small numbers, missed.

It is not clear whether the infected boa constrictors would have eventually progressed to disease and, if so, over what time period. There are many examples of viruses that produce disease only after a long chronic period. For instance, HIV-1 infection typically progresses to AIDS only after years of a mainly subclinical infection. It is possible that a longer chronic phase, secondary infection, stress, or other triggers are necessary for IBD progression in boa constrictors and other less susceptible snakes. Nevertheless, reptarenavirus infection in ball pythons produced neurological signs typical of those associated with IBD, and these viruses remain the leading candidate etiological agent for IBD in all snakes.

One possible explanation for the chronic subclinical infection in boa constrictors is that they are a reservoir host for reptarenaviruses in the wild (23). Boa constrictors (family Boidae) are native to the Americas, and ball pythons (family Pythonidae) are found in Africa. It is possible that reptarenaviruses have coevolved with and adapted to their natural reptile hosts in the Americas, as is the case for the New World lineage of mammal-infecting arenaviruses (11, 12, 24). Additional sampling of wild snakes will address this possibility.

It is possible that reptarenavirus genotype influences clinical outcome. Indeed, a large number of genetically diverse reptarenaviruses have been described, and it is possible that some reptarenaviruses would produce disease outcomes different from those observed here. For instance, a reptarenavirus not studied here might cause disease in boa constrictors but not ball pythons. It would therefore be imprudent to extrapolate from these results to all reptarenaviruses. Nevertheless, prior studies have observed a strong connection between snake species and the IBD clinical course, whereas no connection between reptarenavirus genotype and clinical outcome has been noted to date (1, 2, 7, 9, 14, 15). In addition, in our experiment, ball pythons infected with different reptarenavirus genotypes exhibited similar clinical signs: both python L infected with GoGV and python M infected with GoGV and snake 37 virus displayed severe neurological signs. We identified a subset of the inoculated genome segment genotypes in python M's brain (S6/L3), indicating that the genotype combinations S2/L2 and S6/L3 produced similar disease.

One of our motivations for coinfecting python M with GoGV and snake 37 virus was to begin to investigate the phenomenon of multiple reptarenavirus infection (14, 15). This phenomenon is surprisingly common in captive snakes and is characterized by intrahost virus populations composed of multiple distinct viral genotypes and by an imbalance between the numbers of S and L segment genotypes in a single infection. For instance, the snake 37 virus inoculum was composed of 3 genetically distinct reptarenavirus segments: S6, L3, and L21 (the GoGV genome is simply S2 and L2). This virus was isolated from an infected boa constrictor, and the 3 segments replicate as an ensemble in culture (14). In our survey of reptarenavirus diversity, S6 was by far the most prevalent S segment genotype, both at a population level and in individual snakes, suggesting that it may be outcompeting the S segments of other genotypes (14). That S6 was the only S genotype detected in the brain of coinfected ball python M supports this suggestion, but larger studies will be necessary for a more conclusive investigation of this intriguing phenomenon.

Despite the name IBD, the connection between inclusions and disease is clearly not straightforward. It is now well established that reptarenavirus infection produces the inclusions associated with IBD (8, 10, 22). However, inclusions do not necessarily indicate disease and disease does not require inclusions. Inclusions can be found in apparently healthy snakes, and in infected ball pythons, viral nucleoprotein was cytoplasmic but was not found in inclusion bodies. We speculate that inclusion bodies may accumulate slowly, and given the rapid disease onset in ball pythons, inclusions may not have had enough time to form. Indeed, the granular appearance of cytoplasmic anti-NP staining in python tissues is reminiscent of the staining pattern observed in boa JK cells shortly after infection (8). Thus, reptarenavirus infection produces inclusions and inclusion body disease, but inclusions *per se* are not pathognomonic for IBD, despite assertions to that effect (16).

This study has implications for the control of IBD in captive snake populations. Our data suggest that large quantities of virus may be shed in feces, urates, and skin. Thus, infected boas could be actively transmitting virus during a chronic and subclinical period, confounding disease control and quarantine measures. It would be prudent to separate boa constrictors and pythons until the boa constrictors have been confirmed by molecular methods to be free of reptarenaviruses, which have now been unambiguously linked to disease in ball pythons.

## MATERIALS AND METHODS

### Ethics statement.

This study, including protocols for the care, handling, and infection of animals, was approved by the University of California, Davis, Institutional Animal Care and Use Committee (IACUC protocol 17450).

### Preparation of virus stocks.

Virus stocks for inoculation were prepared by infecting boa constrictor JK cells with low-passage-number stocks of Golden Gate virus (GoGV) (8) or snake 37 virus, the virus population isolated from snake 37 (14). Ten-centimeter-diameter dishes of infected JK cells were cultured as described previously (8). Supernatant was collected at 4, 7, 10, and 13 days postinfection and stored at −80°C. Viral RNA was purified from the supernatant using a Zymo Research viral RNA kit and screened for viral RNA levels by qRT-PCR as described below. Supernatants with the highest viral RNA levels were pooled and clarified by centrifugation at 930 × *g* for 5 min at room temperature. Clarified supernatants were filtered through a 0.22-μm-pore-size filter and underlaid with a 30% sucrose cushion in a centrifuge bottle (catalog number 355618; Beckman Coulter). Viruses were concentrated by ultracentrifugation at 140,000 × *g* in a Thermo Fisher Scientific F50L-8x39 rotor for 2 h at 4°C. The supernatant was decanted, and the pellet was resuspended in 1 to 2 ml phosphate-buffered saline (PBS). Aliquots were stored at −80°C and titrated using a fluorescent focus assay as described previously (25).

### Snake husbandry and monitoring.

Three adult boa constrictors (Boa constrictor; one male control, one male infected, one female infected) and four adult ball pythons (Python regius; two female controls, one female infected, one male infected) were used for this study. Control and infected snakes were housed in separate buildings and were handled independently, and each animal had its own tank and supplies. Following procurement, the snakes were allowed to acclimate to their housing for 3 weeks prior to the start of the study. Whole blood was collected for overall health assessment and for arenavirus RNA by qRT-PCR prior to inclusion in the study. During the acclimation and study periods, the snakes were monitored twice daily for overall health. Animals that exhibited any abnormal neurological signs (star gazing, head tilt, tongue flicking), gastrointestinal signs (regurgitation, diarrhea, constipation), or respiratory clinical signs, that repeatedly declined food, or that exhibited steady body weight loss were to be euthanized.

### Liver and lung biopsy samples.

After the acclimation period, liver and lung biopsy samples were collected while the snakes were under isoflurane anesthesia. The snakes were again anesthetized, and surgical lung and liver biopsy samples were collected at 4 and 8 months postinoculation. Biopsy samples were examined histopathologically and for reptarenavirus RNA by qRT-PCR and metagenomic NGS.

### Snake inoculation and blood sample collection.

Several weeks after the initial biopsy samples were collected, mock or experimental infections were administered by intracardiac injection of the viral inoculum in 200 μl PBS while the snakes were under general anesthesia (the anesthetic protocol was identical to that described above for collection of biopsy samples). We chose this route of infection because the natural routes of reptarenavirus transmission in the wild remain unknown and because prior studies have shown that reptarenaviruses replicate in blood cells (20). Thereafter, every 14 days for the 1st 3 months, 0.3- to 0.5-ml whole-blood samples were collected via cardiocentesis with manual restraint, using a 25-gauge needle on a 1- or 3-ml syringe. A minimum of 3 blood smears were made, and the remaining blood was collected in lithium-heparin tubes and stored at −80°C until testing. At 2 months, 3 months, and 18 months, an additional 0.25-ml whole-blood sample was collected into a K_2_ EDTA tube for a complete blood count (and biochemistry panel at 18 months). After 3 months, blood was collected monthly for 9 months and then every 3 months during the second year of the study.

### Euthanasia and postmortem examination.

The snakes were euthanized using 100 mg/kg of body weight pentobarbital, administered by the intracardiac route, while the snakes were under isoflurane anesthesia either after the exhibition of clinical signs or at the end of the study. A full postmortem examination was performed.

Sections of brain, spinal cord, trachea, lung, liver, kidney, spleen, pancreas, adrenal glands, gonads, heart, tonsil, and complete gastrointestinal tract were collected and placed in 10% buffered formalin, fixed, processed as 5-μm sections, and stained with hematoxylin and eosin (H&E). A second identical set of tissues was immediately flash frozen in liquid nitrogen and stored at −80°C.

### Immunofluorescence staining and imaging.

Paraffin-mounted slides were deparaffinized with the following series of 3-min washes: mixed xylenes (2 times), 50% mixed xylenes to 50% ethanol, 100% ethanol (2 times), 95% ethanol, 70% ethanol, 50% ethanol, and deionized water (2 times). Antigen retrieval by a 30-min incubation at 99°C in EDTA buffer (1 mM EDTA with 0.05% Tween 20) followed. The slides were then rinsed three times with deionized water and washed in 50 mM Tris, pH 7.6, 150 mM NaCl (Tris-buffered saline [TBS]) containing 0.025% Tween 20 for 5 min (2 times). Permeabilization was done in PBS with 0.1% Triton X-100 for 5 min, followed by 5-min washes in TBS with 0.05% Tween 20 (TBS-T) (4 times). After the slides were washed, they were blocked in blocking buffer (5% donkey serum, 1% bovine serum albumin [BSA] in TBS) for 20 min and incubated overnight at 4°C in antinucleoprotein primary antibody (8) at a 1:1,000 dilution in TBS with 1% BSA, followed by washing in TBS-T for 5 min (4 times). Donkey anti-rabbit immunoglobulin conjugated with Alexa Fluor 488 (catalog number A-21206; Thermo Fisher Scientific) secondary antibody was then applied at a 1:400 solution in TBS with 1% BSA for 30 min at room temperature in the dark. Finally, the slides were washed in TBS-T (4 times) and mounted using Prolong antifade mounting reagent with DAPI (catalog number P36931; Thermo Fisher Scientific). Imaging was performed on a Zeiss Axio Scan microscope using a ×20 lens or on a Nikon Ti microscope with a Andor Zyla 4.2 scientific grade complementary metal-oxide semiconductor (sCMOS) spinning-disk camera with a ×100 lens. Image processing was done using the Zeiss software Zen Microscopy and ImageJ software (26).

### RNA extraction.

RNA was extracted from solid tissue samples, feces, urates, and shed skin samples as previously described (14). Purified and DNase-treated RNA samples were resuspended in 50 μl of RNase- and DNase-free water and quantified fluorometrically. To extract RNA from blood, 250 μl of whole blood was added to a 2-ml tube containing 1 ball bearing and 1 ml of TRIzol reagent (Invitrogen) and homogenized using a TissueLyser tissue disrupter (Qiagen) for 2 to 3 min at 30 Hz. Homogenized samples were mixed with 200 μl of chloroform, and the mixture was incubated at room temperature for 2 min and centrifuged for 10 min at 12,000 × *g* at 4°C. The aqueous phase was mixed with 450 μl cold isopropanol, and the mixture was incubated at 4°C for 1 h. Samples were centrifuged for 10 min at 12,000 × *g* at 4°C, and the supernatant was decanted. Precipitated RNA was washed with 1 ml of 75% ethanol and incubated for 10 min at 4°C. RNA was pelleted by centrifugation for 10 min at 12,000 × *g* at 4°C. Ethanol was removed and the pellet was allowed to air dry before it was resuspended in 80 μl of RNase- and DNase-free water. Samples were treated with 20 units of DNase I (NEB) and incubated at 37°C for 30 min. To the DNase-treated samples, 100 μl of a phenol-chloroform-isoamyl alcohol mixture (125:24:1, pH 4.3) was added, and the mixture was incubated at room temperature for 15 min and then centrifuged for 3 min at 12,000 × *g* at 4°C. The aqueous phase was transferred to a new 1.5-ml tube, and RNA was precipitated using a GlycoBlue coprecipitant protocol (Ambion) with a prolonged incubation step of 30 min. Samples were DNase treated twice, followed by phenol-chloroform extraction and coprecipitation with GlycoBlue coprecipitant.

### Illumina sequencing and data analysis.

Sequencing libraries were prepared from RNA and analyzed as previously described (14).

### qRT-PCR.

RNA (500 ng) was added to 1 μl of 250 μM random hexamer oligonucleotide, and the mixture was incubated at 65°C for 5 min. Master mix was added to final concentrations of 1× reaction buffer, 5 mM dithiothreitol, 1.25 mM (each) deoxynucleoside triphosphates (dNTPs), and 0.5 μl of SuperScript III reverse transcriptase (Life Technologies). The reaction mixtures were incubated for 5 min at 25°C, then for 30 to 60 min at 42°C, and then for 15 min at 70°C. cDNA was diluted to 100 μl (1:10) in water. Each quantitative PCR (qPCR) mixture contained 5 μl diluted cDNA, 1× Hot FirePol mix Plus (Solis Biodyne), and 0.5 μM each primer. qPCRs were run on a Roche LightCycler 480 instrument with thermocycling conditions of 15 min at 95°C and 40 cycles of 10 s at 95°C, 12 s at 60°C, and 12 s at 72°C. Viral RNA levels were calculated using linearized plasmid standard curves. The primers (primer sequences) used for qPCR were MDS-558 (TTGATCTTCAGTCAGGACTTTACG) and MDS-559 (RACCTTGGTTCCACTGCTG) for S6; MDS-530 (ATGAGTGAGYCGACCTCCATAG) and MDS-531 (CRAGTGCCAATGATGTAAGAGAA) for L3; MDS-538 (CCTCCATTGGCCTAACAACT) and MDS-539 (CAAGAGCAAGAGAGGTCAGAGAG) for L21; MDS-554 (CGGTGAATCCTAGTGAGGAG) and MDS-555 (CTACCTTGGACCCACTGGAA) for S2; MDS-532 (CGRCTCCACCGCCATT) and MDS-533 (GAGTGCTAGTGARGAAAGAGATCC) for L2; MDS-785 (TGTCACAATGATGACCCTCAA) and MDS-786 (GGGCCAGTGATGAGAGAGAC) for L13; and MDS-921 (AATATCTGCCCCATCAGCTG) and MDS-923 (GTTTTCCAAGAGCGTGATCC) for GAPDH. In some instances, Sanger sequencing was used to verify the qRT-PCR products.

### Accession number(s).

Sequencing data have been deposited in the Sequence Read Archive under BioProject accession number PRJNA383000.

## Supplementary Material

Supplemental material

## References

[1] Schumacher J, Jacobson E, Homer B, Gaskin J 1994 Inclusion body disease in boid snakes. J Zoo Wildl Med 25:511–524.

[2] Chang L-W, Jacobson ER 2010 Inclusion body disease, a worldwide infectious disease of boid snakes: a review. J Exot Pet Med 19:216–225. doi:10.1053/j.jepm.2010.07.014.

[3] Schilliger L, Selleri P, Frye FL 2011 Lymphoblastic lymphoma and leukemic blood profile in a red-tail boa (Boa constrictor constrictor) with concurrent inclusion body disease. J Vet Diagn Invest 23:159–162. 21217051 10.1177/104063871102300131

[4] Summa NM, Sanchez-Migallon Guzman D, Hawkins M, Grosset C, Chen V, Goldsmith D, Keel K, Woolard K, Young A, Bucy D, Steffey M 2015 Tracheal and colonic resection and anastomosis in a boa constrictor (Boa constrictor) with T-cell lymphoma. J Herpetol Med Surg 25:87–99. doi:10.5818/1529-9651-25.3.87.

[5] Schilliger L, Rossfelder A, Bonwitt J, Di Girolamo N, Rival F, Gandar F, Selleri P, Nicolier A 2014 Antemortem diagnosis of multicentric lymphoblastic lymphoma, lymphoid leukemia, and inclusion body disease in a boa constrictor (Boa constrictor imperator). J Herpetol Med Surg 24:11–19. doi:10.5818/1529-9651-24.1.11.

[6] Jacobson ER 2007 Infectious diseases and pathology of reptiles: color atlas and text. CRC/Taylor & Francis, Boca Raton, FL.

[7] Wozniak E, McBride J, DeNardo D, Tarara R, Wong V, Osburn B 2000 Isolation and characterization of an antigenically distinct 68-kd protein from nonviral intracytoplasmic inclusions in Boa constrictors chronically infected with the inclusion body disease virus (IBDV: Retroviridae). Vet Pathol 37:449–459. doi:10.1354/vp.37-5-449. 11055868

[8] Stenglein MD, Sanders C, Kistler AL, Ruby JG, Franco JY, Reavill DR, Dunker F, Derisi JL 2012 Identification, characterization, and in vitro culture of highly divergent arenaviruses from boa constrictors and annulated tree boas: candidate etiological agents for snake inclusion body disease. mBio 3:e00180-12. doi:10.1128/mBio.00180-12. 22893382 PMC3419519

[9] Bodewes R, Kik MJL, Raj VS, Schapendonk CME, Haagmans BL, Smits SL, Osterhaus ADME 2013 Detection of novel divergent arenaviruses in boid snakes with inclusion body disease in The Netherlands. J Gen Virol 94:1206–1210. doi:10.1099/vir.0.051995-0. 23468423

[10] Hetzel U, Sironen T, Laurinmäki P, Liljeroos L, Patjas A, Henttonen H, Vaheri A, Artelt A, Kipar A, Butcher SJ, Vapalahti O, Hepojoki J 2013 Isolation, identification, and characterization of novel arenaviruses, the etiological agents of boid inclusion body disease. J Virol 87:10918–10935. doi:10.1128/JVI.01123-13. 23926354 PMC3807292

[11] Radoshitzky SR, Bào Y, Buchmeier MJ, Charrel RN, Clawson AN, Clegg CS, DeRisi JL, Emonet S, Gonzalez J-P, Kuhn JH, Lukashevich IS, Peters CJ, Romanowski V, Salvato MS, Stenglein MD, de la Torre JC 2015 Past, present, and future of arenavirus taxonomy. Arch Virol 160:1851–1874. doi:10.1007/s00705-015-2418-y. 25935216

[12] Buchmeier MJ, de la Torre JC, Peters CJ 2013 Arenaviridae, p 1283–1303. *In* Knipe DM, Howley PM, Cohen JI, Griffin DE, Lamb RA, Martin MA, Racaniello VR, Roizman B (ed), Fields virology, 6th ed Lippincott Williams & Wilkins, Philadelphia, PA.

[13] Salvato MS, Clegg JCS, Buchmeier MJ, Charrel RN, Gonzalez JP, Lukashevich IS, Peters CJ, Romanowski V Arenaviridae, p 715–723. *In* Virus taxonomy. Classification and nomenclature of viruses. Ninth report of the International Committee on Taxonomy of Viruses. Elsevier Academic Press, San Diego, CA.

[14] Stenglein MD, Jacobson ER, Chang L-W, Sanders C, Hawkins MG, Guzman DS-M, Drazenovich T, Dunker F, Kamaka EK, Fisher D, Reavill DR, Meola LF, Levens G, DeRisi JL 2015 Widespread recombination, reassortment, and transmission of unbalanced compound viral genotypes in natural arenavirus infections. PLoS Pathog 11:e1004900. doi:10.1371/journal.ppat.1004900. 25993603 PMC4438980

[15] Hepojoki J, Salmenperä P, Sironen T, Hetzel U, Korzyukov Y, Kipar A, Vapalahti O 2015 Arenavirus coinfections are common in snakes with boid inclusion body disease. J Virol 89:8657–8660. doi:10.1128/JVI.01112-15. 26041290 PMC4524219

[16] Keller S, Hetzel U, Sironen T, Korzyukov Y, Vapalahti O, Kipar A, Hepojoki J 2017 Co-infecting reptarenaviruses can be vertically transmitted in boa constrictor. PLoS Pathog 13:e1006179. doi:10.1371/journal.ppat.1006179. 28114434 PMC5289648

[17] Aqrawi T, Stöhr AC, Knauf-Witzens T, Krengel A, Heckers KO, Marschang RE 2015 Identification of snake arenaviruses in live boas and pythons in a zoo in Germany. Tierarztl Prax Ausg K Klientiere Heimtiere 43:239–247. doi:10.15654/TPK-140743. 26109078

[18] Hellebuyck T, Pasmans F, Ducatelle R, Saey V, Martel A 2015 Detection of arenavirus in a peripheral odontogenic fibromyxoma in a red tail boa (Boa constrictor constrictor) with inclusion body disease. J Vet Diagn Invest 27:245–248. doi:10.1177/1040638714562825. 25776548

[19] Abba Y, Hassim H, Hamzah H, Ibrahim OE, Ilyasu Y, Bande F, Mohd Lila MA, Noordin MM 2016 In vitro isolation and molecular identification of reptarenavirus in Malaysia. Virus Genes 52:640–650. doi:10.1007/s11262-016-1345-7. 27142080

[20] Chang L-W, Fu A, Wozniak E, Chow M, Duke DG, Green L, Kelley K, Hernandez JA, Jacobson ER 2013 Immunohistochemical detection of a unique protein within cells of snakes having inclusion body disease, a world-wide disease seen in members of the families Boidae and Pythonidae. PLoS One 8:e82916. doi:10.1371/journal.pone.0082916. 24340066 PMC3858296

[21] Hepojoki J, Kipar A, Korzyukov Y, Bell-Sakyi L, Vapalahti O, Hetzel U 2015 Replication of boid inclusion body disease-associated arenaviruses is temperature sensitive in both boid and mammalian cells. J Virol 89:1119–1128. doi:10.1128/JVI.03119-14. 25378485 PMC4300630

[22] Chang L, Fu D, Stenglein MD, Hernandez JA, DeRisi JL, Jacobson ER 2016 Detection and prevalence of boid inclusion body disease in collections of boas and pythons using immunological assays. Vet J 218:13–18. doi:10.1016/j.tvjl.2016.10.006. 27938703

[23] Mandl JN, Ahmed R, Barreiro LB, Daszak P, Epstein JH, Virgin HW, Feinberg MB 2015 Reservoir host immune responses to emerging zoonotic viruses. Cell 160:20–35. doi:10.1016/j.cell.2014.12.003. 25533784 PMC4390999

[24] Charrel RN, de Lamballerie X, Emonet S 2008 Phylogeny of the genus Arenavirus. Curr Opin Microbiol 11:362–368. doi:10.1016/j.mib.2008.06.001. 18602020

[25] Koellhoffer JF, Dai Z, Malashkevich VN, Stenglein MD, Liu Y, Toro R, Harrison SJ, Chandran K, DeRisi JL, Almo SC, Lai JR 2014 Structural characterization of the glycoprotein GP2 core domain from the CAS virus, a novel arenavirus-like species. J Mol Biol 426:1452–1468. doi:10.1016/j.jmb.2013.12.009. 24333483 PMC3951589

[26] Schneider CA, Rasband WS, Eliceiri KW 2012 NIH Image to ImageJ: 25 years of image analysis. Nat Methods 9:671–675. doi:10.1038/nmeth.2089. 22930834 PMC5554542

