# Supplementary material for: Differential Disease Susceptibilities in Experimentally Reptarenavirus-Infected Boa Constrictors and Ball Pythons
Source: J Virol. 2017 Jul 12;91(15):e00451-17. doi: 10.1128/JVI.00451-17 (PMC5651717; doi:10.1128/JVI.00451-17)
Supplement: Supplemental material [file supp_91_15_e00451-17__index.html]

Differential Disease Susceptibilities in Experimentally Reptarenavirus-Infected Boa Constrictors and Ball Pythons — Supplemental material 

# Differential Disease Susceptibilities in Experimentally Reptarenavirus-Infected Boa Constrictors and Ball Pythons

## Supplemental material

- Supplemental file 1 -

  Legend to Movie S1

  PDF, 42K
- Supplemental file 2 -

  Movie S1 (Reptarenavirus-infected ball python displaying neurologic signs typical of inclusion body disease.)

  AVI, 14M
