# Supplementary material for: Differential Disease Susceptibilities in Experimentally Reptarenavirus-Infected Boa Constrictors and Ball Pythons
Source: J Virol. 2017 Jul 12;91(15):e00451-17. doi: 10.1128/JVI.00451-17 (PMC5651717; doi:10.1128/JVI.00451-17)
Supplement: Supplemental material [file JVI.00451-17_zjv999182753s1.pdf]

Supplemental Movie Legend

Supplemental Movie 1: Reptarenavirus-infected ball python displaying neurologic signs typical of inclusion body disease. Uninfected ball pythons exhibiting normal behavior by recovering quickly after being placed on their backs.
